# Catatonia; A Case Study and Literature Review

**DOI:** 10.1192/j.eurpsy.2022.1988

**Published:** 2022-09-01

**Authors:** A. Ogunlela, H. Raai

**Affiliations:** SBH Health System, Department Of Psychiatry And Behavioral Health, New York, United States of America

**Keywords:** Catatonia, Lorazepam challenge, schizophrénia, ECT

## Abstract

**Introduction:**

Catatonia is a state of apparent unresponsiveness to external stimuli in a person who is awake. More common in patients with unipolar major depression or bipolar disorder. Common signs: immobility, rigidity, mutism, posturing, excessive motor activity, stupor, negativism, staring, and echolalia. We will discuss a case of a 23 year old male with schizophrenia presented with catatonia and decompensation of his schizophrenia in the context of medication non-compliance. We will discuss findings from litrature pertaining to catatonia and treatment strategies.

**Objectives:**

- To discuss catatonia, its incidence in different psychiatric disorders. - To discuss literature pertaining to catatonia. - To discuss different treatment strategies

**Methods:**

- Case study

**Results:**

- Signs of catatonia: immobility, mutism, withdrawal and refusal to eat, staring, negativism, posturing, rigidity, waxy flexibility/catalepsy, stereotypy, echolalia, or echopraxia, verbigeration. - Diagnosis: Clinical, Lorazepam challenge. Bush-Francis Catatonia Rating Scale (BFCRS) - BFCR scale is used as the screening tool. If 2 of the 14 are positive, prompts further evaluation and completion of the remaining 9 items. - Differential Diagnosis include; Neuroleptic Malignant Syndrome, Serotoninergic Syndrome, Malignant Hyperthermia, Akinetic Mutism, Delirium, Parkinson’s disease. - Lorazepam can be scheduled at interval doses until the catatonia resolves. - ECT in combination with benzodiazepines is used to treat malignant catatonia. - Possible complications are Physical trauma, malignant catatonia (autonomic instability, life-threatening), dehydration, pneumonia, pressure ulcers due to immobility, muscle contractions, DVT, PE

**Conclusions:**

Psychiatrists need to be diligent in evaluating patients with Catatonia for other comorbid psychiatric conditions, addressing these conditions and conducting a thorough assessment and prompt treatment.

**Disclosure:**

No significant relationships.

